# Cigarette smoking prevalence and associated factors among college students, Amhara, Ethiopia

**DOI:** 10.11604/pamj.2021.40.170.24413

**Published:** 2021-11-19

**Authors:** Animut Takele Telayneh, Mihretie Gedefaw, Dessalegn Haile, Samuel Derbie Habtegiorgis, Daniel Shitu Getahun, Yilkal Tafere, Moges Agazhe Assemie, Wubetu Woyraw, Habtamu Temesgen, Nakachew Mekonnen

**Affiliations:** 1 Department of Public Health, Debre Markos University, Debre Markos, Ethiopia,; 2 Department of Nursing, Debre Markos University, Debre Markos, Ethiopia,; 3 Department of Human Nutrition, Debre Markos University, Debre Markos, Ethiopia

**Keywords:** Cigarette smoking, college of teachers’ education, Ethiopia

## Abstract

**Introduction:**

tobacco is the only legal drug that kills many of its users when used exactly as intended by the manufacturers. It is estimated that of the 1.1 billion smokers worldwide, nearly 80% of them live in low and middle-income countries. This trend increases in college and university students with most smokers starting to smoke during adolescent. The aim of this study is to assess cigarette smoking prevalence and associated factors among a select group of college of teachers´ education students.

**Methods:**

a cross-sectional study was conducted. Multistage sampling was used to select 605 study participants from across the eight departments of the Injibara College of Teachers´ Education. Each subject was selected by simple random sampling technique after proportional allocation to each class. EpiData version 4.2 was used for data entry and Stata version 14 was used for data cleaning and analysis. Variables with p-value < 0.2 in bi-variable analysis were selected for multi-variable analysis. Adjusted odds ratio (AOR) with 95% confidence interval (CI) was reported to show the strength of association.

**Results:**

the current prevalence of cigarette smoking is 6.8% amongst the Injibara College of Teachers´ Education students. Males [AOR: 2.84 (95% CI: 1.13, 7.14)], divorced marital status [AOR: 7.27 (95% CI: 1.23, 42.85)], food source in hostel [AOR: 11.62 (95% CI 3.23, 41.71)] and exposure to family/other smokers [AOR: 6.17 (95% CI: 2.17, 16.06)] were statistically significant factors for cigarette smoking.

**Conclusion:**

the prevalence of cigarette smoking was relatively low. Male, marital status, source of food, and exposure to family/other smokers were identified associated factors. Policy makers and health regulatory body are strongly encouraged to consider this evidence and the associated factors for smoking in their efforts to develop and implement tobacco control laws.

## Introduction

Cigarette smoking is one of the leading causes of preventable morbidity and premature mortality globally [1-4]. According to World Health Organization (WHO) definition a smoker is someone who smokes any tobacco product, either daily or occasionally. Tobacco is the only legal drug that kills many of its users when uses exactly as intended by the manufacturers [5]. Globally, tobacco use has become an escalating public health concern [2]. It is estimated that 1.1 billion peoples smoke cigarettes [5,6]. Tobacco kills at least half of long-term smokers and is related to more pooled deaths each year than Human Immune Virus/Acquired Immune Deficiency Syndrome, Tuberculosis, and Malaria [6]. Around 8 million projected death annually are attributed to cigarette smoking with among this more than 80% accounted from Low and Middle Income Countries (LMICs) [7,8]. Direct smokers accounts for approximately 6 million death per each year and 600,000 are attributed to second hand smoke [7,9]. At the current rate, smoking related deaths are projected to exceed 8 million in 2030 [1,5]. Much of the morbidity and mortality associated with tobacco use relate to cancers, chronic lower respiratory obstructive conditions and cardiovascular disease [10]. Cigarette smoking is an important risk factor for all-causes of vascular disease mortality [11].

Cigarette smoking has declined significantly in developed countries; however, it is increasing in LMICs [2,12]. The LMICs are facing an increasing burden of tobacco related diseases. Africa, particularly sub-Saharan Africa including Ethiopia is facing increasing tobacco use [13]. The magnitude of tobacco-related deaths in Africa increased from 150,000 reported deaths in 1990 to over 215,000 in 2016 which representing about 70% increase in mortality [9]. The highest number of deaths occurred in Eastern Africa and the lowest was in Central Africa [9].

Many smokers are initiated into the habit between the ages of 13 and 15 years [2]. Adolescent smokers are a source of public health importance because many are at risk of future morbidity later in life [1,5,14]. In 2000, globally about a third of population aged 15 years and older was used tobacco; whereas this declined to 24.9% by 2015. By gender during this same time period the rate of male tobacco users went from three to four times that of female users [7]. In 2016, an estimated one-fifth of males and one-third of females globally were exposed to secondhand smoke [10].

Previous studies conducted with school adolescents, college, and university students of current cigarette smoking revealed that 13.7% in Nigeria [2], 30.1% in Cameron with gender variation [4] and 10.7% (21.09% of male and 0.02% female) [15] in Pakistan. Other findings showed that 20.20% reported having smoked and 12.94% were current smokers in India private medical students [16]. The overall prevalence of tobacco smoking was 60.2% in Bangladesh [17], 14.7% in Malaysia [18] and (24.8% males and 9.1% females) in Saudi Arabia [19]. Similarly, reported current smoker rates included 12.4% in a Yemen community college [20], 5.7% in Ilorin, Nigeria [21], and 11% amongst Iranian university students [22]. Studies conducted with Ethiopian undergraduate university students showed the prevalence rates of current smokers as 14.5% in Jigjiga [23], 20.6% in Hawassa [24], 19.17% in Della [25] and 1.8% in Addis Ababa [26].

Many students will start smoking at college or university. Previous findings have that use of tobacco was affected by age, male sex, place of residence, fraternity, type of family, being unmarried, family income, pocket money, alcohol use, ever khat use, year of study, having friend and family member who smokes, tension reduction, pleasure, number of sticks smoked daily, and peer pressure [2,4,14,18-22,27].

The WHO developed the WHO Framework Convention on Tobacco Control (WHO-FCTC) which articulates a range of strategies to control the tobacco epidemic. Included in the WHO-FCTC were recommendations such as price and taxation to reduce demand, non-price measures to reduce demand through effective legislation which are protective (such as reducing exposure to smoke), regulator (such as content and risk disclosures), and educative (such as awareness and advertising restrictions) [7,8]. Despite these efforts, tobacco epidemic reduction efforts remained inefficient globally [8]. In Ethiopia, implementation status of tobacco legislation has focused on the prohibiting or restricting of sales of flavored (e.g. menthol) products or to persons under 18 years of age; banning of all form of tobacco advertisement (including, promotion and sponsorship) with mandatory, health hazard package and labeling; as well as, increased taxation and establishing of smoking free areas [13].

Previously studies in Ethiopia among college and university students [23,24] and at the population level through the Ethiopian demographic and health Survey [28] revealed deviation of cigarette smoking prevalence across regions. The problem in Ethiopia remains unacceptably high, which reinforces the imperative for evidence-informed laws and policies, especially as these addresses the framework to establish an integrated strategy for college and, university students and the community at large. Therefore, this study aims to determine the prevalence of cigarette smoking and its associated factors among college of teachers´ education students in the effort to address this gap in the evidence to strengthen future legislation and policy.

## Methods

**Study design and eligibility criteria:** a cross-sectional study design was conducted from April 28^th^, 2019 to May 2^nd^, 2019. The study was conducted in Northwest Amhara Region, which hosts three colleges of teachers´ education at Injibara, Finote Selam, and Debre Markos. Injibara Collage of Teachers´ Education (ICTE) was selected purposively because it is situated within a special group of community called “Agew” at Injibara town. It is located 435 kilometer (km) away from Addis Ababa and 118 km from Bahir Dar. Injibara town has an estimated total population of 43,777 as according to the 2019 town administrative office report. The ICTE was established in July 2009 and currently served for 1,692 students in 8 departments. All regular ICTE students in 2019 were considered as source population.

**Figure 1:**
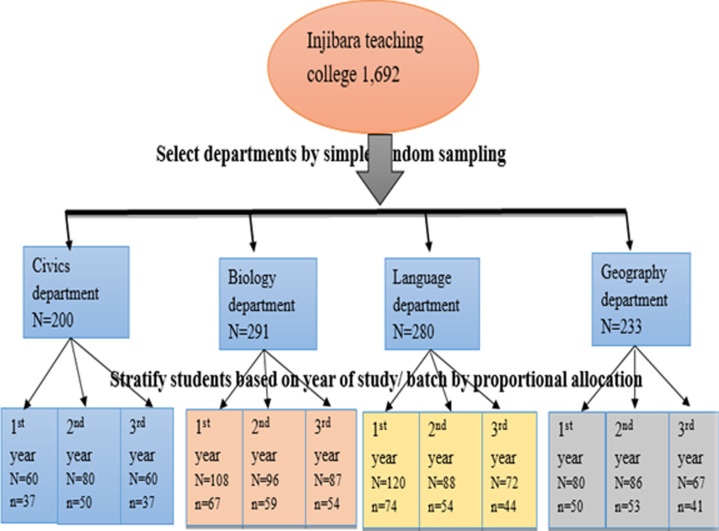
sample size allocation for cigarette smoking among college students at Injibara College of Teachers’ Education, Amhara, 2019

**Sampling procedure:** sample size was determined using a single population proportion formula taking 20.6% [24] proportion of study conducted in Hawassa University students by considering 95% CI, 4% level of precision, 1.5 design effect, and 5% non-response rate yielding a sample of 620. Multi-stage sampling technique was used across the eight departments selecting four departments (with a total number of 1,004 students) through a simple random lottery sampling method. Then, the selected departments were stratified by year of study. Finally, the required numbers of study subjects were selected from each year of study by size proportional allocation (Figure 1).

**Study variables:** the outcome variable was current cigarette smoking (yes/no) which was considered in terms of sociodemographic and economic factors (i.e. age, sex, religion, ethnicity, residence, source of food, source of pocket money, monthly pocket money, batch/year, marital status, with whom currently living, and department), and predisposing and other risk factors (number of sticks smoked daily, duration of smoking behavior, peer pressure, family member smoker, presence of friend as smoker, poor academic performance, and relief from tension and stress).

### Operational definition

**Ever smoker:** referred to as a student who had ever tried to smoked cigarettes during his/her life time.

**Current user:**a student who had smoked cigarettes on one or more days in the last one month (30 days).

**Data collection procedures and quality assurance:** the data collection tool was prepared in English, and then translated in to Amharic for data collection. All data were then translated to English to maintain consistency and coherent for analysis. Data were collected through using self-administered questionnaires. Three public health fourth year students´ data collectors and one master of public health supervisor were participated. Data quality was maintained through training of data collectors and the supervisor. Close supervision was maintained during the entire data collection period. All filled questionnaires were checked for completeness, clarity and consistency. Any missed or unfilled data was corrected immediately during the data collection period. Finally all collected data was reviewed and check for completeness before data entry.

**Data processing and analysis:** EpiData version 4.2 was used for data entry and Stata version 14 used for data cleaning and analysis. Bi-variable analysis for selection of variables with P-value less than 0.2 for multi-variable analysis with P-value less than 0.2 was done. Binary logistic regression was used to identify associated factors with cigarette smoking. Multicollinearity was assessed for multi variable binary logistic regression and model fitness was checked through the Hosmer Lemeshow test. All necessary descriptive data was presented using texts, frequency tables and graphs. The p-value <0.05 with 95% CI corresponding AOR were used to identify statistically associated factors for cigarette smoking.

**Ethics approval and consent to participate:** ethical approval was obtained from Ethics Committee of Debre Markos University Health Science College. The formal letter from the Health Science College was submitted to ICTE. Participants in the study were engaged on a voluntary basis and oral informed consent was taken for each participating student and confidentiality of data was maintained by excluding the name of participants recorded in the questionnaires. Study participants were informed that the research process has no any form of incentives, force, and does not bring any risks.

## Results

**Socio-demographic characteristics:** a total of 605 respondents participated with a 97.6% of response rate. The majority (564 (93%)) of study participants were orthodox religion followers. Only 96 (15.9%) of study participants were 25 years and above, 48 (8%) were married and 177 (29.3%) were currently living with family (Table 1).

**Table 1:** socio-demographic characteristics of cigarette smoking among college students at Injibara College of Teachers´ Education, Amhara, 2019

Characteristics of respondent	Frequency (N)	Percent (%)
**Sex**		
Male	334	55
Female	271	45
**Age in years**		
15-19	113	18.7
20-24	396	65.4
≥25	96	15.9
**Religion**		
Orthodox	564	93
Muslim	36	6
Protestant	5	1
**Ethnicity**		
Amhara	435	72
Awi	170	28
**Marital status**		
Single	543	90
Married	48	8
Divorced	14	2
**Have boy/girlfriend (557)**		
No	397	71.3
Yes	160	28.7
**With whom currently live**		
Alone	144	23.8
Family	177	29.3
Others*	284	46.9

Note: *relative, classmate, and friends

**Socio-economic and educational status of respondents:** among study participants almost one fifth (19.8%) were civics department and 212 (35%) were second year students. For the majority (87.8%), the source of monthly income was from parents/family members, with 174 (28.8%) receiving above 1,000 Ethiopian birr (ETB) monthly pocket money (Table 2).

**Table 2:** socio-economic and educational status of cigarette smoking among college students at Injibara College of Teachers’ Education, Amhara, 2019

Characteristics of respondent	Frequency (N)	Percent (%)
**Department**		
Language	168	27.8
Biology	174	28.8
Geography	143	23.6
Civics	120	19.8
**Batch/year of study**		
First-year	219	36.2
Second year	212	35
Third year	174	28.8
**Source of income**		
Parents/family members	531	87.8
Self-pocket	68	11.2
Other*	6	1
**Monthly pocket money**		
≤1000 ETB	431	71.2
>1000 ETB	174	28.8
**Relationship with family**		
Not good	15	2.5
Good	590	97.5
**Source of food**		
Parent/family	98	16.2
Self-cook	467	77.2
Hotel guest	40	6.6

Note: *relatives and friends

**Figure 2:**
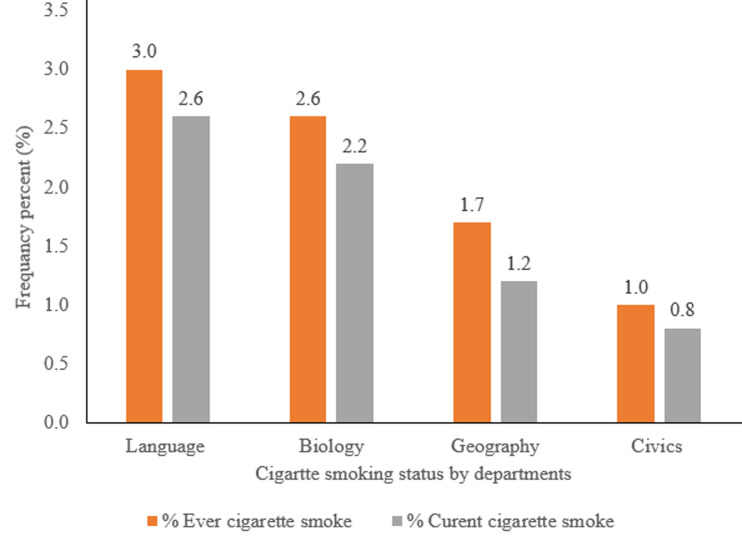
comparison of cigarette smoking prevalence among students between departments at Injibara College of Teachers’ Education, Amhara, 2019

**Figure 3:**
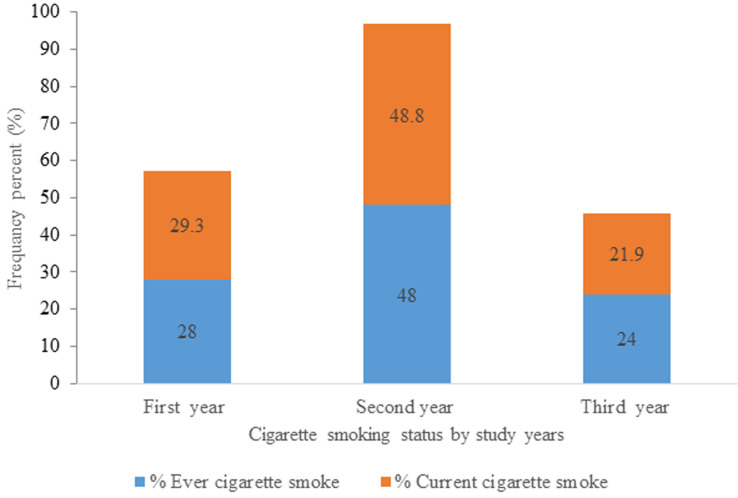
proportion of cigarette smoking based on year of study/batch among students at Injibara College of Teachers’ Education, Amhara, 2019

**Cigarette smoking status and triggering factors of respondent:** the prevalence of current cigarette smokers was 41 (6.8%) with a gender difference of male at 5.6% and at female 1.2%; whereas 50 (8.3%) reported ever cigarette smoking with male (6.8%) and female (1.5%). Amongst ever smokers 33 (66%) study subjects started smoking at college, with 25 and 8 of them were started at first and second years respectively. Sixty six (11%) of study subjects were exposed to smoke from family/other smokers. Only 17 (26%) of study subjects were providing support to quit smoking for other smokers. In this study the prevalence of cigarette smoking was high in language department enrollees and being second year students (Table 3, Figure 2, Figure 3).

**Table 3:** cigarette smoking triggering factors among college students at Injibara College of Teachers´ Education, Amhara, 2019

Character of respondent	Frequency (N)	Percent (%)
**Time to start smoking (50)**		
Elementary	5	10
Secondary	6	12
Preparatory	6	12
College	33	66
**Is there any triggering factor for smoking (50)**		
No	14	28
Yes	36	72
**What are possible triggering reasons (36)**		
Peer pressure	14	39
Refreshment	6	17
Family member smoking	7	19
Academic stress	9	25
**Exposure to family/other smokers**		
No	539	89
Yes	66	11
**Whom you contact smoker (66)**		
Parent	7	10.6
Sister/brother	11	16.7
Other*	48	72.7
**What you feel about the family/other smokers (66)**		
Nothing	20	30.3
Disgust	36	54.6
Encouraged	10	15.1

Note: *classmate, relative, and friend

**Patterns of smoking and related health problems:** among current smokers, more than half (56%) had smoked for more than 12 months and 61% smoked more than 6 sticks per day. Half (51.2%) of study subjects were trying to quit smoking. In the current study, 12, 7, and 2 current smokers reported respiratory, gastro-intestinal, and mental illness related to smoking respectively (Table 4).

**Table 4:** patterns of smoking and related health problems of cigarette smoking among students at Injibara College of Teachers´ Education, Amhara, 2019

Characteristics of respondent	Frequency (N)	Percent (%)
**Duration of smoking among current smokers (41)**		
≤12 month	18	44
>12 month	23	56
**Number of sticks smoked per day (41)**		
≤6 sticks	25	61
>6 sticks	16	39
**Ever worried about being smoking (41)**		
No	8	19.5
Yes	33	80.5
**Are you trying to quit (41)**		
No	20	48.8
Yes	21	51.2
**Which health problem will occur due to quit smoking (41)**		
Depression	17	41.5
Behavioral change	8	19.5
Missing class	7	17
More than one health problem	9	22
**Health problem related smoking (41)**		
No	20	48.8
Yes	21	51.2
**Treatment was given for health problem (21)**		
No	8	38
Yes	13	62

**Associated factors of cigarette smoking:** in bi-variable analysis, sex, religion, marital status, department, year of study/batch, with whom currently live, monthly pocket money, relationship with family, source of food, and exposure to family/other smokers´ variables were selected for multi-variable analysis. Finally, in multi-variable analysis only variables including; male (AOR: 2.84 (95% CI: 1.13, 7.14)), divorced marital status (AOR: 7.27 (95% CI: 1.23, 42.85)), food source in hostel (AOR: 11.62 (95% CI 3.23, 41.71)), and exposure to family/other smokers (AOR: 6.17 (95% CI: 2.17, 16.06)) were selected as significantly associated factors for cigarette smoking (Table 5).

**Table 5:** bi-variable and multi-variable analysis of cigarette smoking among students at Injibara College of Teachers´ Education, Amhara, 2019

Variable	Frequency N (%)	COR (95% CI)	P-value	AOR (95% CI)	P-value
**Sex**					
Male	234(55.2)	4.27(1.86, 9.80)*	0.001	2.84(1.13, 7.14)*	0.026
Female	271(44.8)	1		1	
**Marital status**					
Single	543(89.8)	1		1	
Married	48(7.9)	0.61(0.14, 2.62)	0.509	0.73(0.11, 4.98)	0.746
Divorced	14(2.3)	3.84(1.02, 14.38)*	0.046	7.27(1.23, 42.85)*	0.028
**Batch/year of study**					
First-year	219(36.2)	1.06(0.44, 2.58)	0.893	2.00(0.64, 6.21)	0.233
Second-year	212(35)	1.90(0.85, 4.31)	0.119	1.80(0.62, 5.22)	0.277
Third year	174(28.8)	1		1	
**Monthly pocket money**					
≤1000 birr	431(71.2)	1		1	
>1000 birr	174(28.8)	2.82(1.48, 5.34)*	0.001	1.19(0.47, 2.97)	0.705
**Relationship with family**					
Not good	15(2.5)	3.63(0.98, 13.42	0.053	3.16(0.66, 14.95)	0.146
Good	590(97.5)	1		1	
**Source of food**					
In family	98(16.2)	1		1	
In self-cook	467(77.2)	0.50(0.19, 1.34)	0.174	0.38(0.13, 1.11)	0.078
In hotel	40(6.6)	15.3(5.46, 43.05)**	0.001	11.62(3.23, 41.71)**	0.001
**Exposure to family/other smokers**					
No	539(89)	1		1	
Yes	66(11)	5.80(2.89, 11.66)**	0.001	6.17(2.37, 16.06)**	0.001

Significant level *<0.05, **<0.001

## Discussion

In this study, the prevalence of current smoking was 6.8% (95% CI: 5.02, 9.08) with higher rates in male than female students. This finding is similar study reported with 6.3% in Cameron [4] and 5.7% in Nigeria Ilorin university students [21]. The similarity may be due to large sample size of previous studies. This finding is lower than college and university students reported in Pakistan 10.7% [15], and Iran (11% Kerman College and 21% Semnan Province) [22,29], 12.94% in Rajasthan [16], 13.7% in Nigeria [2], 14.7% in Malaysia [30], 60% in Dhaka Bangladesh [17], 54% in India [31], 14% in Western Saudi Arabia [19], 12.4% in Yemen [20], and 10.5% in Iraq [27]. Furthermore, previous studies in Ethiopia university students indicated prevalence of 11.3% in Wollo [32], 11.8% in Adigrat [33], 14.5% Jigjiga [23], 19.17% in Dilla [25], and 20.6% in Hawassa [24]. Such differences may be due to the socio-cultural, demography, and religious aspects of cigarette smoking across the country and within the respective nations.

In this study, use of cigarette smoking is quite low as compared to previous studies which may be due to negative connotations of smoking by the special community called “Agew” in the region, as well as sample size difference. This study finding was higher than 4% in school students of Tanzania [34], 3% urban setting in Nigeria secondary schools [35] and 1.8% in Addis Ababa medical university students [26]. This discrepancy may be due to difference of study setting conducted at urban and early age enrolling of study participants in Tanzania and Nigeria.

In this study, sex of study participant is associated with cigarette smoking: male students are almost 3 fold at risk to cigarette smoked compared to female students (AOR: 2.84 (95% CI 1.13, 7.14)). This finding was consistent with most of previous studies conducted in cigarette smoking among students in Iran, Rajasthan, Cameron, Iraq, Malaysia, Western Saudi Arabia, Yemen, Nigeria, and Ethiopia [2,4,16,19,20,22,27,30,34]. The lower smoking association may be due to low intention for use by female students and social attached stigma of female smokers in the community at large.

Students who were divorced were 7.27 times more likely to cigarette smoke compared to single counter parts (AOR: 7.27 (95% CI: 1.23, 42.85)). This finding is not coincide with other similar study conducted in Iraq university unmarred students are more risk [27]. The disagreement in findings may relate to practice of early marriage at most regions of country. For instance, early married is more likely result in divorce this may cause socially isolation and psychological depression. Hence, divorced students are perceived socially unacceptable which increases the probability of engaging in risky behaviors including cigarette smoking in order to relieve tension and stress.

Students whose food sources at the hostel were 11.62 fold at risk to cigarette smoke compared to those whose primary food source was within family (AOR; 11.62(95% CI: 3.23, 41.71)). Students who had daily food in hostels were exposed to a variety of risk behaviors such as attending night clubs, engage in unsafe sex, use of substance like (alcohol, cigarette and khat), and haphazard way of life.

Students those exposed to family/other smokers were more than 6 times more likely to cigarette smoke compared to their counter-parts with no sources exposure (AOR: 6.17 (95% CI: 2.37, 16.06)). This finding is in line with similar studies conducted in Iran, Cameron, Pakistan, Bangladesh, Nigeria, Tanzania and Ethiopia [2,4,15,17,22,25,29,33,34]. This trend may be related to these exposures leading to the individual considered as a secondary smoker.

## Conclusion

The prevalence of current cigarette smoking was relatively low in this study. Male sex, divorced marital status, primary source of food as hostel, and exposure to family/others smokers were identified risk factors for cigarette smoking. Policy makers, health regulators and all other responsible bodies must consider these risk factors in their efforts to promptly and efficiently implement tobacco controlling legislation to end tobacco smoking. Future research is required to further explore each of these risk factors.

**Limitation of the study:** the respondents may be unable to recall all previous exposure or practices in relation to cigarette smoking during this data collection time. This study´s findings, difficult to generalize to all college and university students in Ethiopia because it is pertain to only ICTE students.

**Availability of data and material:** the datasets used and/or analyzed during the current study are available from the corresponding author on reasonable request.

### What is known about this topic

- Cigarette smoking is a current and persistent public health problem;
- Younger individuals are more at risk for uptake cigarette smoking;
- Despite, the existed controlling lows of cigarette smoking the implementation remains unacceptable.

### What this study adds

- In this finding, the current cigarette prevalence was found to be 6.8%;
- Study participants being male, marital status, food source as the hostel, and exposure to family/others smokers are identified risk groups to cigarette smoke;
- This finding show, the most (48.8%) of second year students were current cigarette smoker compared to first- and third-year students.
